# Impedance Spectroscopy Study of Charge Transfer in the Bulk and Across the Interface in Networked SnO_2_/Ga_2_O_3_ Core–Shell Nanobelts in Ambient Air

**DOI:** 10.3390/s24196173

**Published:** 2024-09-24

**Authors:** Maciej Krawczyk, Ryszard Korbutowicz, Patrycja Suchorska-Woźniak

**Affiliations:** Faculty of Electronics, Photonics and Microsystems, Wrocław University of Science and Technology, Wybrzeże Wyspiańskiego 27, 50-370 Wroclaw, Poland; ryszard.korbutowicz@pwr.edu.pl (R.K.); patrycja.suchorska-wozniak@pwr.edu.pl (P.S.-W.)

**Keywords:** metal oxide, nanowires, nanotapes, core–shell, heterostructures, impedance spectroscopy, nanofibers, nanostructures

## Abstract

Metal oxide core–shell fibrous nanostructures are promising gas-sensitive materials for the detection of a wide variety of both reducing and oxidizing gases. In these structures, two dissimilar materials with different work functions are brought into contact to form a coaxial heterojunction. The influence of the shell material on the transportation of the electric charge carriers along these structures is still not very well understood. This is due to homo-, hetero- and metal/semiconductor junctions, which make it difficult to investigate the electric charge transfer using direct current methods. However, in order to improve the gas-sensing properties of these complex structures, it is necessary to first establish a good understanding of the electric charge transfer in ambient air. In this article, we present an impedance spectroscopy study of networked SnO_2_/Ga_2_O_3_ core–shell nanobelts in ambient air. Tin dioxide nanobelts were grown directly on interdigitated gold electrodes, using the thermal sublimation method, via the vapor–liquid–solid (VLS) mechanism. Two forms of a gallium oxide shell of varying thickness were prepared via halide vapor-phase epitaxy (HVPE), and the impedance spectra were measured at 189–768 °C. The bulk resistance of the core–shell nanobelts was found to be reduced due to the formation of an electron accumulation layer in the SnO_2_ core. At temperatures above 530 °C, the thermal reduction of SnO_2_ and the associated decrease in its work function caused electrons to flow from the accumulation layer into the Ga_2_O_3_ shell, which resulted in an increase in bulk resistance. The junction resistance of said core–shell nanostructures was comparable to that of SnO_2_ nanobelts, as both structures are likely connected through existing SnO_2_/SnO_2_ homojunctions comprising thin amorphous layers.

## 1. Introduction

Semiconducting metal oxides (MOXs) are commonly used as gas-sensitive materials for the detection of a wide variety of both reducing and oxidizing gases due to their low manufacturing costs, miniature size, and ease of use [1]. Metal oxides in the form of fibrous nanostructures such as nanowires, nanotapes, nanobelts, nanotubes, and electrospun nanofibers are promising gas-sensitive materials [2,3]; such potential can be attributed to their large surface-area-to-volume ratio, mechanical flexibility [4], and dynamic response to variations in the composition of ambient air, as well as the good long-term stability of their sensing parameters [5]. Fibrous nanostructures can be formed into spatial, open networks that facilitate the diffusion of the gaseous atmosphere deep into the structure. In addition, due to their small cross-sections, surface phenomena have a significant impact on their bulk electrical parameters.

A lack of selectivity, limited sensitivity, and insufficient detection thresholds hinder the application of MOX fibrous nanostructures in many fields; one example of such fields is that of non-invasive health diagnostics, in which trace amounts of disease markers are detected in exhaled breath [6]. Researchers have attempted to improve the sensitivity and selectivity of MOX fibrous nanostructures through the synthesis of core–shell heterostructures. In these structures, two dissimilar materials with different work functions are brought into contact to form a coaxial heterojunction. The formation of the junction causes the flow of electric charge carriers between the two materials. As a result, the layers of both accumulated and depleted charge are formed in the immediate vicinity of the heterojunction [7,8], which has a significant influence on the transport of electric charge carriers along the structures. In addition, the shell of the structure that is exposed to the ambient atmosphere will affect the gas-sensing properties of the heterostructure. However, existing studies have shown that, in a network of interconnected MOX fibrous nanostructures, the homojunctions between the structures are largely responsible for the modulation of resistance in response to changes in the composition of ambient air [9,10]. The importance of the junction between core–shell heterostructures in the context of their gas-sensing properties is often overlooked. In order to improve these gas-sensing properties in a deliberate way, it is necessary to establish a good understanding of electric charge transfer as it occurs in an ambient atmosphere. Therefore, we must investigate how the shell material impacts the charge transfer in the bulk and across the interface between the structures.

Over the past two decades, many efforts have been made to study the effect of the shell on the gas-sensing properties of networked MOX core–shell fibrous nanostructures [11,12,13,14,15,16,17,18,19,20,21]. The numerous homo-, hetero-, and metal/semiconductor junctions make it difficult to study these structures. As their complexity increases, the characterization of the coaxial heterojunction and the junction between the structures becomes yet more challenging. At present, direct current methods are most often used to characterize the electrical and gas-sensing parameters of MOX fibrous core–shell structures. However, DC methods are not well-suited to the task of separating bulk parameters from those of the junction between the structures and the metal/semiconductor junction. As a result, we often encounter difficulties in determining the direction of the flow of charge carriers between the core and shell material [14,22]. This is further complicated by the fact that the value of the work function of the core and the shell materials can differ significantly from the values commonly accepted for bulk materials [13,14]. Because of this, researchers’ opinions are divided on whether accumulation layers may form when defects in the heterojunction region are present [15,16,17]. Furthermore, the cause of the significant increase in the resistance of n–n-type core–shell fibrous nanostructures in ambient air (which is not observed in surface-decorated structures [23]) has not been fully explored [12,14,21].

It is thought that these difficulties can be overcome by utilizing impedance spectroscopy [21]. To date, a number of studies have explored MOX gas-sensitive fibrous nanostructures using this method [9,24,25]. However, to the authors’ knowledge, impedance spectroscopy has not yet been used to examine how the shell material impacts charge transfer in core–shell fibrous structures in ambient air.

This study is focused on the use of impedance spectroscopy to investigate the impact of the Ga_2_O_3_ shell on the charge transfer in the bulk and across the junction between SnO_2_/Ga_2_O_3_ core–shell nanobelts in ambient conditions. SnO_2_ and Ga_2_O_3_ were selected as they are both n-type semiconductors with gas-sensitive properties. Networked SnO_2_/Ga_2_O_3_ core–shell nanobelts were synthesized directly on gold electrodes via a thermal sublimation method reliant on the vapor–liquid–solid mechanism. Two types of gallium oxide shells that differed in thickness were synthesized by halide vapor-phase epitaxy (HVPE). The impedance spectra of the SnO_2_ nanobelts and the SnO_2_/Ga_2_O_3_ core–shell structures were studied in ambient air at 189–768 °C. We were then able to present an electrical equivalent model of the studied structures. The bulk resistance of core–shell nanobelts was reduced due to the accumulation of the electrons in the SnO_2_ core. At temperatures above 530 °C, the thermal reduction in SnO_2_ and the associated decrease in its work function caused the electrons to flow from the accumulation layer towards the Ga_2_O_3_ shell. This resulted in an increase in the bulk resistance. Both kinds of structures were likely connected through existing SnO_2_/SnO_2_ homojunctions made of thin amorphous layers, which we can deduce from the lack of significant impact that the Ga_2_O_3_ shell had on the junctions’ resistance in ambient air.

## 2. Materials and Methods

**Figure 1:**
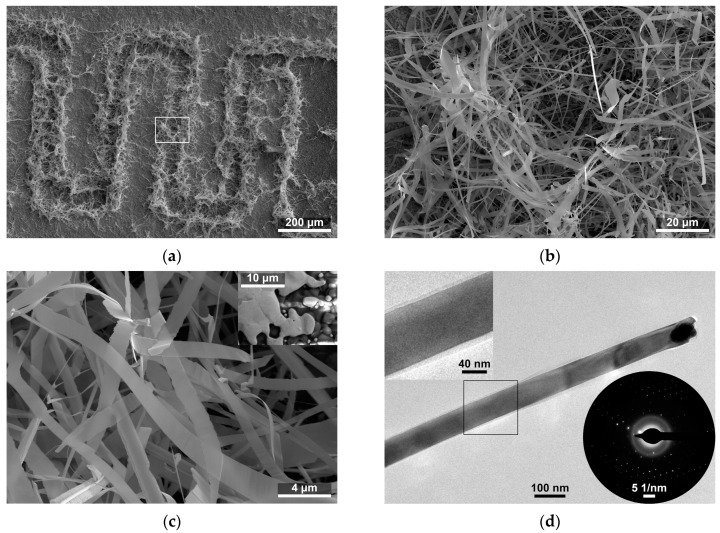
(**a**) Scanning electron microscope image of SnO_2_ nanobelts grown on interdigitated electrodes; and (**b**,**c**) enlarged image of the microstructure of SnO_2_ nanobelts. The inset shows the edge of the Au electrode before synthesis; and (**d**) Transmission electron microscope image of SnO_2_ nanobelts. The inset in the upper corner shows an enlarged view of the amorphous layer on the surface of the nanobelt. The inset in the lower corner shows the selective area diffraction pattern of the imaged nanobelt.

Three pairs of gold (8846-G, ESL Europe, Reading, UK) interdigitated electrodes (IDEs) were made with thick-film technology on a 96% Al_2_O_3_ alumina ceramic substrate (CeramTec, Plochingen, Germany) with dimensions of 25 mm × 2.5 mm × 0.25 mm. The dimensions of a single electrode were 150 ± 10 μm × 590 ± 10 μm, and the distance between the electrodes was 70 ± 5 μm (Figure 1a).

The synthesis of networked SnO_2_ nanobelts was performed on as-prepared interdigitated electrodes in a tubular resistive furnace (Nabertherm, Lilienthal, Germany). A strip of metallic tin was placed in a quartz boat, and the substrate with electrodes was placed at a distance of 5 mm downstream from the Sn strip. No additional growth catalyst was used. Two mixing streams were supplied to the inlet of the furnace: 500 mL/min of dry nitrogen and 400 mL/min of humidified nitrogen that flowed through a bubbler filled with deionized water kept at near-boiling temperature. Synthesis was carried out at near-atmospheric pressure as the furnace’s outlet was open to the ambient atmosphere. The quartz boat was inserted into the preheated furnace in a swift movement. Synthesis was carried out at 950 °C for a duration of 105 min. After the synthesis, the quartz boat was quickly removed from the hot furnace and cooled at room temperature in ambient air.

The synthesis of the Ga_2_O_3_ shell was carried out in a three-zone resistance furnace. The temperature in the growth zone was 840 °C for the SnO_2_/Ga_2_O_3_ core–shell sample denoted CS840 and 1000 °C for the sample denoted CS1000. The temperature in the chlorination zone was 860 °C for both samples. The synthesis was carried out at atmospheric pressure in an open quartz reactor. The carrier gas was nitrogen flowing at 6000 mL/min mixed with synthetic air flowing at 1000 mL/min. A quartz boat with liquid gallium was placed in the chlorination zone. Hydrogen chloride, diluted with 250 mL/min of nitrogen, flowed through the quartz boat at a rate of 30 mL/min when synthesizing sample CS840 and 10 mL/min when synthesizing sample CS1000. The alumina ceramic substrate with SnO_2_ nanobelts was placed on a quartz boat in the growth zone. The CS840 shell was synthesized for 10 min, and the CS1000 shell was synthesized for 15 min, after which the boat was quickly removed from the furnace and cooled to room temperature in the ambient air.

After the synthesis, two alumina ceramic substrates of the same size were bonded together: one with as-prepared nanostructures, and the other with a meander-shaped platinum heater (5545,ESL Europe, Reading, UK), which was made with thick-film technology. Pd–Ag paste (9635-B, ESL Europe, Reading, UK) was used as the adhesive layer. After the firing of the adhesive layer, the substrates were permanently bonded together. The electrical connections were soldered to the gold leads of the electrodes and the heater.

The microstructure and the crystal structure were studied with a scanning electron microscope (SEM), SU6600 (Hitachi, Hitachinaka, Japan); a Tecnai G2 20 X-TWIN (FEI, Hillsboro, OR, USA) high-resolution transmission electron microscope (HRTEM); and an Empyrean (Malvern Panalytical, Malvern, UK) X-ray diffractometer (XRD). CuKα radiation was used. A cross-section of the core–shell nanostructures was examined with an SEM/FIB Helios G4 electron microscope (FEI, Hillsboro, OR, USA) equipped with an energy-dispersive (EDS) detector (Bruker, Billerica, MA, USA). The structures were etched with a xenon ion beam.

The electrical measurements were carried out in ambient air with a relative humidity of 30–50% and a temperature of 20 °C. The temperature of the substrate was controlled in the range of 189–768 °C by the Pt heater powered by an E3632A DC power supply (Agilent Technologies, Santa Clara, CA, USA).

Impedance spectra were measured with a 1260 impedance analyzer (Solartron Analytical, Franborough, UK) controlled by ZPlot 3.5f software (Scribner, Southern Pines, NC, USA). Six-inch RG-58U coaxial cables were connected to the samples in a four-terminal pair configuration [26]. The impedance spectra were measured in the range of 32 MHz to 100 Hz with 1 V_RMS_ sinusoidal voltage. In order to eliminate the influence of the substrate and the wires on the measured impedance spectra, the results were subjected to open–short–load compensation [26]. The impedance spectra of the shorted and open interdigitated electrodes were measured at the same temperature as the studied nanostructures. A precision 100 Ω resistor was used as the known load. The measurements of the resistor that were taken on the test fixture connected directly at the terminals of 4294 A impedance analyzer (Agilent Technologies, Santa Clara, CA, USA) were used as the reference value of the load. The electrical equivalent circuit was fitted in ZView 4.0c software (Scribner, Southern Pines, NC, USA). The current–voltage characteristics were measured with a 1287A potentiostat (Solartron Analytical, Franborough, UK), controlled with CorrWare 3.1c software (Scribner, Southern Pines, NC, USA).

## 3. Results

### 3.1. Structural Characterization

**Figure 2:**
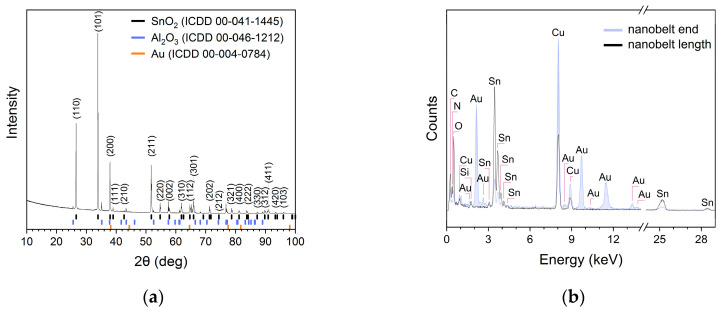
(**a**) X-ray diffractogram of SnO_2_ nanobelts; and (**b**) chemical composition examined along the length and at the end of the nanobelt shown in Figure 1d.

The growth of the SnO_2_ fibrous nanostructures occurs primarily at the edges of the gold interdigitated electrodes (Figure 1a). An image of the edge of the Au electrode before synthesis is shown in the inset in Figure 1c. The structures resemble nanobelts and nanotapes (hereafter collectively referred to as nanobelts), which form a dense network of connection in the space between the electrodes (Figure 1a–c). High-magnification observations made with HRTEM have shown that Au or Au–Sn alloy droplets are present at the ends of some structures (Figure 1d), and, herein, their composition was identified via EDX (Figure 2b). We found thin amorphous layers on the surface of the nanostructures (Figure 1d). The presence of the amorphous layers was also confirmed by the halo visible on the selective area electron diffraction (SAED) patterns (Figure 1d inset). However, distinctive dots on the pattern indicate that—apart from the surface amorphous layers—the studied nanostructures are monocrystalline.

The peaks in the X-ray diffractogram (Figure 2a) of the prepared nanobelts were matched to the standard data of rutile-type SnO_2_ powders (ICCD 00-41-1445) with an elemental unit cell size of a = 0.474 nm and c = 0.319 nm. The remaining peaks in the diffractogram can be attributed to the alumina substrate and gold. The EDX analysis of the chemical composition (Figure 2b) showed that the SnO_2_ nanobelts were composed of tin and oxygen atoms.

**Figure 3:**
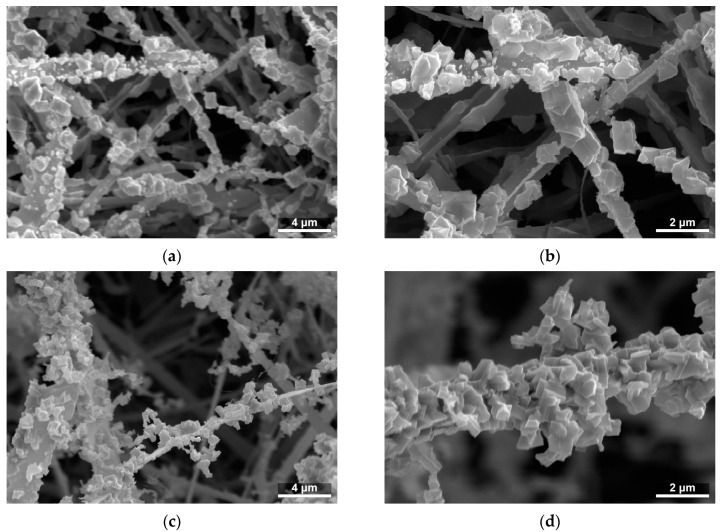
SEM images of the microstructure of SnO_2_/Ga_2_O_3_ core–shell nanobelts: (**a**) CS840; (**b**) enlarged image of CS840; (**c**) CS1000; and (**d**) enlarged image of CS1000.

The Ga_2_O_3_ shells prepared on SnO_2_ nanobelts featured a granular structure and a developed surface (Figure 3). Through the deliberate selection of the temperature, synthesis time, and hydrogen chloride flow rate, two shells of differing thickness were produced.

**Figure 4:**
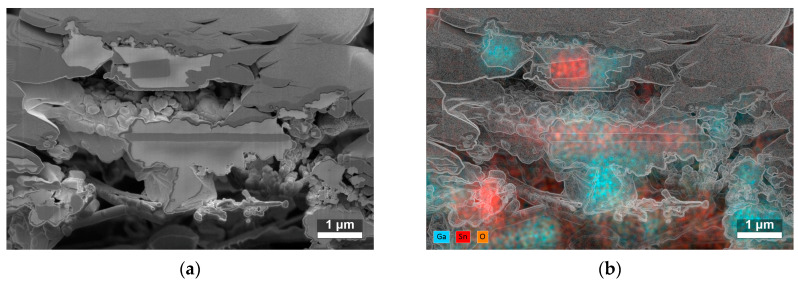
(**a**) SEM image of the cross-section of two SnO_2_/Ga_2_O_3_ core–shell fibrous structures with shells synthesized at 840 °C. In the upper part of the image, the sputtered Pt layer before ion beam etching is visible (for more detail, see Figure S1 in the Supplementary Materials), and, in the lower part, the alumina substrate can be seen. (**b**) Corresponding map of the atomic composition of the cross-section; the edges of the SEM image are overlaid to aid visualization.

The cross-sections of two SnO_2_/Ga_2_O_3_ core–shell structures synthesized at 840 °C (Figure 4a) showed that the nanobelts and nanotapes were covered by a granular shell with a developed surface. The cores of these structures can be locally exposed, especially the edges of the nanotapes. The EDS analysis of the atomic composition of the cross-sections (Figure 4b) indicated that they were composed of tin, oxygen, and gallium atoms (see Figure S1 in the Supplementary Materials for more detail).

### 3.2. Impedance Spectroscopy

#### 3.2.1. Impedance Spectra and the Electrical Equivalent Circuit

**Figure 5:**
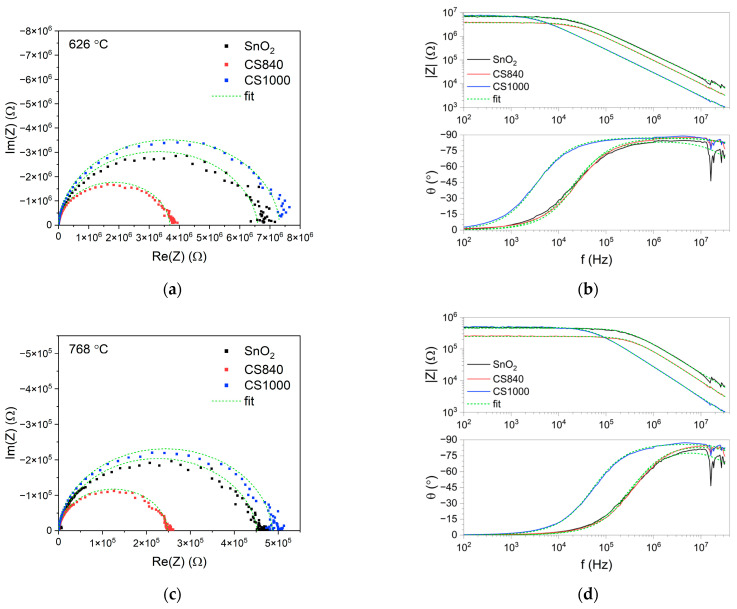
Complex impedance and Bode plots of the tested structures, as measured at (**a**,**b**) 626 °C and (**c**,**d**) 768 °C.

The points on the complex impedance plot formed a depressed semicircle (Figure 5a,c). The impedance modulus in the low-frequency range decreased exponentially as a function of temperature (for more detail, see Figure S2 in the Supplementary Materials). The value of the impedance modulus in the low-frequency range differed slightly between the tested structures (Figure 5b,d). These differences were more pronounced at temperatures above 530 °C, at which point the impedance modulus of CS840 was lower than that of the other samples. In the high-frequency range, the impedance modulus of the SnO_2_ nanobelts was higher than that of the core–shell structures.

In the high frequency range, the theta angle slowly decreased with the frequency (Figure 5b,d). The dip in the theta angle at around 20 MHz we can attribute to the reflection of the high-frequency signal from the end of the transmission line: the SnO_2_ nanobelts are much more susceptible than the core–shell structures. This shows the mismatch between the high-frequency impedance of the tested structures and the impedance of the cables.

**Figure 6:**
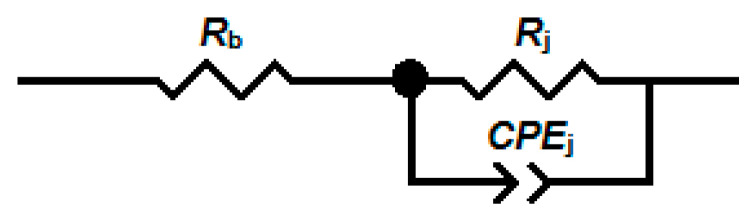
The electrical equivalent circuit fitted to the impedance spectra. *R*_b_ is the bulk resistance, *R*_j_ is the resistance of junctions between the nanobelts, and *CPE*_j_ is the constant-phase element.

The electrical equivalent circuit (Figure 6) was fitted to the impedance spectra. The impedance of the constant-phase element (*CPE*) used to model the junctions between the nanobelts is given by the following formula [27]:

(1) $$Z_{CPE}=\frac{1}{Q{\left(j\omega \right)}^{\alpha }}=\frac{1}{Q\omega^{\alpha }}\left(\cos\frac{\alpha \pi }{2}-j\sin\frac{\alpha \pi }{2}\right),$$

where *ω* = 2π*f* is the angular frequency in rad/s, *α* is a non-ideality factor taking values from 0 to 1, and the value of the parameter *Q* of the constant-phase element *CPE* has the unit F/s^(1−*α*)^.

The fit of the electrical equivalent circuit is indicated by the green dashed line in Figure 5.

#### 3.2.2. The Bulk and Junction Electrical Parameters

The electrical parameters of the nanostructures (the bulk resistance *R*_b_, the resistance of the junctions between the structures *R*_j_, and the junction capacitance *C*_j_) were determined using the parameters of the electrical equivalent circuit.

**Figure 7:**
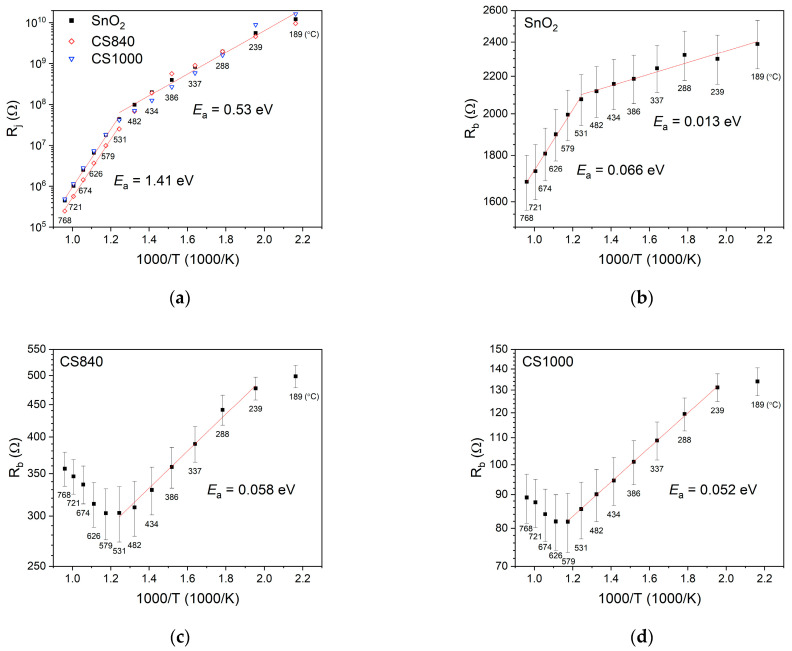
Arrhenius plots of (**a**) the resistance of junctions between the structures; (**b**) the bulk resistance of SnO_2_ nanobelts; (**c**) the bulk resistance of CS840; and (**d**) the bulk resistance of CS1000.

The junction resistance *R*_j_ of the SnO_2_ nanobelts and the core–shell structures was similar (Figure 7a). The junction resistance decreased exponentially as a function of temperature. At temperatures below approximately 530 °C, the energy of activation for all of the studied structures, as determined using the Arrhenius equation, was about 0.53 eV. Above this temperature, the resistance of the junctions decreased rapidly, and the determined energy of activation was about 1.41 eV. In this temperature range, the junction resistance of CS840 was slightly lower than that of the other structures.

The bulk resistance *R*_b_ of the SnO_2_ nanobelts (Figure 7b) was much smaller than the junction resistance. The bulk resistance of the SnO_2_ nanobelts decreased as a function of temperature. The activation energy determined at temperatures below approximately 530 °C was about 13 meV. At temperatures above approximately 530 °C, the energy of activation was about 66 meV.

The bulk resistance of SnO_2_/Ga_2_O_3_ core–shell structures (Figure 7c,d) was significantly lower than that of SnO_2_ nanobelts. The changes in the bulk resistance of the core–shell structures were distinct from those of the SnO_2_ nanobelts. The activation energy was 58 meV for CS840 (Figure 7c) and 52 meV for CS1000 (Figure 7d).

The capacitance of the junctions *C*_j_ was then determined as follows [27]:

(2) $$C_{j}={Q_{j}}^{1/\alpha }{\left(\frac{R_{b}R_{j}}{R_{b}+R_{j}}\right)}^{\left(1-\alpha \right)/\alpha }$$

This value remained fairly constant throughout the investigated temperature range. The capacitance of the junctions between the SnO_2_ nanobelts was about 0.8 pF, while the junction capacitance of the core–shell structures was about 1.2 pF for CS840 and about 4.8 pF for CS1000.

## 4. Discussion

The growth of SnO_2_ nanobelts took place mainly at the edges of gold electrodes (Figure 1a). The catalysts for their growth, we assume, were the small islands of Au scattered at the edge of the electrodes (Figure 1c inset), which formed during screen-printing on the alumina ceramic substrate. The growth of nanobelts via the vapor–liquid–solid mechanism resulted in Au or Au–Sn alloy droplets at the ends of the structures (Figure 1d). Thin amorphous layers were found on the surface of the SnO_2_ nanobelts (Figure 1d). On this basis, we can also assume that the SnO_2_ nanostructures formed at the edges of the electrodes were in contact with each other through amorphous SnO_2_/SnO_2_ homojunctions, thus forming a network of connections between the electrodes.

**Figure 8:**
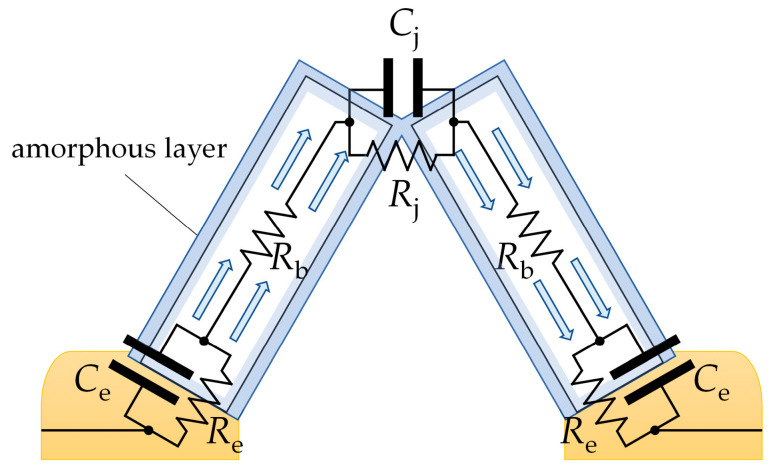
A schematic depiction of electric charge being transported through adjoining SnO_2_ nanobelts. The charge depletion layer is shaded in blue. *R*_e_ and *C*_e_ are the resistance and capacitance of the electrode/semiconductor junction; *R*_b_ is the bulk resistance of the SnO_2_ nanobelt; and *R*_j_ and *C*_j_ are the resistance and capacitance of the junction between the nanobelts.

Figure 8 shows the transport of electric charge through a pair of adjoining SnO_2_ nanobelts, which grow directly on the electrodes. The charge is transported along such an arrangement through the following points:

1. The electrode/semiconductor junction;
2. The conductive channel inside the nanobelts;
3. The junction between the structures.

The existing literature states that, in a network of interconnected SnO_2_ fibrous nanostructures, homojunctions between the nanostructures are largely responsible for changes in the resistance that occur in response to variations in the composition of the ambient atmosphere [9,10]. It is also well-established that, in ambient air, the resistance of a network of interconnected SnO_2_ fibrous nanostructures significantly exceeds the resistance of a single nanostructure [9,28]. Such increased resistance is attributed specifically to the presence of junctions between the nanostructures.

**Figure 9:**
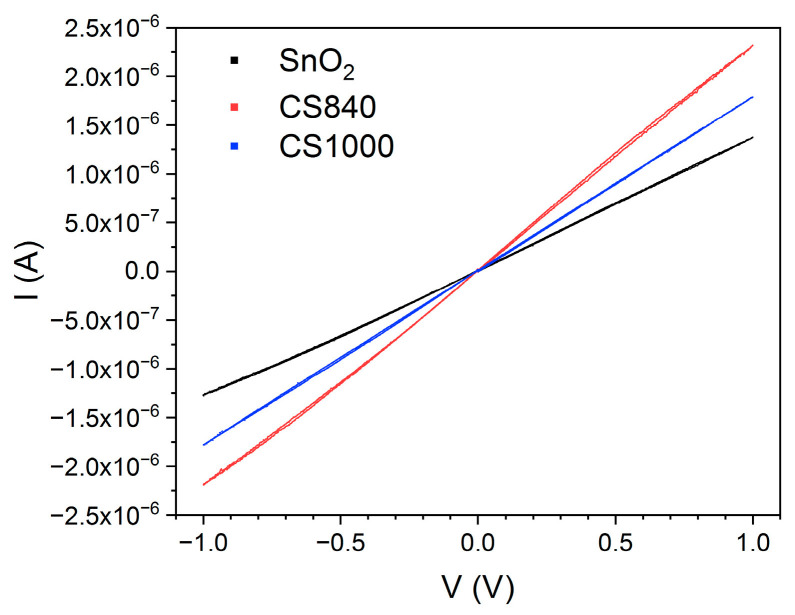
Current and voltage measured at 768 °C.

The impedance modulus of SnO_2_ and SnO_2_/Ga_2_O_3_ core–shell structures measured in the low-frequency range is comparable in value to their resistance, which is determined by their current–voltage characteristics (Figure 9), leading us to conclude that the impedance of the electrode/semiconductor junction does not have a significant effect on the impedance spectra in the investigated frequency range. It is assumed that, for the studied structures, *R*_e_ << *R*_b_ [9]; therefore, the elements *R*_e_ and *C*_e_ (Figure 8) were omitted from the equivalent electrical circuit (Figure 6).

In the case of the studied structures, the electrode/semiconductor junction shows an ohmic character (Figure 9), which may be due to the small difference between the work functions of gold (*Φ*_Au_ = 5.1 eV) and tin dioxide (*Φ*_SnO2_ = 4.9 eV [25]) and the mutual diffusion of the elements in the region of contact. There are reports in the literature regarding the ohmic nature of the metal/semiconductor junction between SnO_2_ fibrous nanostructures, which were synthesized directly on Au/Pt electrodes [10,29]. We can assume that the direct synthesis of SnO_2_ structures on gold electrodes contributes to the formation of a junction of this nature.

Therefore, the impedance spectra (Figure 5) can be interpreted as follows:

(3) $${\left|Z\right|}_{f\to \infty }=R_{b}+R_{e}\;\wedge \;{\left|Z\right|}_{f\to 0}=R_{b}+R_{e}+R_{j}$$

The arrangement of structures between the electrodes consists of many such parallel connections, as presented in Figure 8, which leads to a distribution of time constants. For this reason, a constant-phase element was substituted into the electrical equivalent circuit in place of the junction capacitance *C*_j_ (Figure 6).

The activation energy (*E*_a_ = 0.53 eV), as determined by the characteristics of the SnO_2_ nanobelt junctions (Figure 7a), is in agreement with the value of the surface potential of polycrystalline SnO_2_ grains in the atmosphere of air, while the energy of activation (*E*_a_ = 1.41 eV), as determined at temperatures above 530 °C, is in agreement with the surface potential of SnO_2_ grains in high-vacuum conditions [30]. At high temperatures above approximately 530 °C, as in high-vacuum conditions, oxygen vacancies form on the SnO_2_ surface [31], resulting in an increase in surface potential. These results show that the transport of electric charge between adjoining SnO_2_ nanobelts occurs through amorphous SnO_2_/SnO_2_ homojunctions. The characteristics of junctions in SnO_2_/Ga_2_O_3_ core–shell structures do not differ substantially from those in SnO_2_, indicating that charge transport between core–shell structures also takes place through existing SnO_2_/SnO_2_ homojunctions. The junction resistance of CS840 is, however, noticeably lower at temperatures above 530 °C. Nevertheless, we found its activation to be similar to that of other samples. Presumably, differences in the junction resistance between the samples stem from slight variations in the geometry of the junctions, which cannot be avoided in synthesis involving the vapor–liquid–solid mechanism.

It is accepted that shallow donor levels and deep defects associated with oxygen vacancies exist within the SnO_2_ band gap [31]. The activation energy determined by the bulk characteristics of SnO_2_ nanobelts (Figure 7b) at temperatures below 530 °C is about 13 meV. This value is lower than the typical ionization energies of shallow donors cited in the literature, which are around 30–46 meV [25,31,32]; however, they are in agreement with the activation energy of shallow donors associated with the presence of hydrogen, as reported by King et al. [33]. It is possible for unintentional hydrogen doping to occur during the synthesis of SnO_2_ nanobelts, since the source of oxygen in the synthesis is water vapor. Above 530 °C, the energy of activation determined from the bulk characteristics is about 66 meV, which is in general agreement with the value determined for a single SnO_2_ nanowire in an atmosphere with a low concentration of oxygen (1% O_2_) [25]. This implies that the decrease in the bulk resistance of SnO_2_ nanobelts in this temperature range is due to oxygen vacancies, which, as a result of the thermal decomposition of the material, form not only on the surface but throughout the entire volume of the material.

The combination of SnO_2_ (*Φ*_SnO2_ = 4.9 eV [25]) and Ga_2_O_3_ (*Φ*_Ga2O3_ = 4.0 eV [34]) in a coaxial core–shell heterostructure causes charge carriers to flow through the heterojunction between the two materials. This is due to the difference in their Fermi levels. The flow occurs from the Ga_2_O_3_ shell toward the SnO_2_ core. However, stoichiometric Ga_2_O_3_ is an insulator [35] within which the concentration of free carriers that can flow through the heterojunction into SnO_2_ is relatively low. Other Ga_2_O_3_ structures synthesized in conditions similar to the shells of the studied SnO_2_/Ga_2_O_3_ core–shell nanostructures exhibit low conductivity at temperatures below around 600 °C [36,37]. It is known that the synthesis and annealing of this material in conditions of low oxygen pressure result in the formation of oxygen vacancies and an increase in the conductivity of the material [35]. However, oxygen vacancies form deep donor levels with energies of about 1 eV below the lower edge of the conduction band [38]. The bulk resistance of SnO_2_/Ga_2_O_3_ core–shell nanostructures (Figure 7c,d) is much lower than that of SnO_2_ nanobelts (Figure 7b). In addition, the capacitance of the junctions between the core–shell structures is higher than that of SnO_2_. These results indicate that the flow of the electric charge carriers from the Ga_2_O_3_ shell has occurred and that charge carriers have accumulated in the SnO_2_ core. For a significant number of carriers to flow into the SnO_2_ core, there must be shallow donor states in the band gap of the Ga_2_O_3_ shell that can be ionized at a given experimental temperature. Sn atoms form shallow donor levels in Ga_2_O_3_ with energies of around 60 meV below the lower edge of the conduction band [39]. The ionization energy of such defects is in agreement with the activation energy determined by the bulk characteristics of the core–shell structures of 52–58 meV. It is known that ionization energy decreases with the degree of doping. It is likely that, during high-temperature synthesis, the Ga_2_O_3_ shell is unintentionally doped with Sn atoms that diffuse from the SnO_2_ core. These results suggest that the shell of CS1000, which was synthesized at a higher temperature and for a longer time, may be unintentionally doped to a greater extent.

The work function of the dominant (101) surface of SnO_2_ (Figure 2a) decreases by about 1 eV as a result of the thermal reduction of SnO_2_ [40]. Therefore, the increase in the bulk resistance of SnO_2_/Ga_2_O_3_ core–shell structures at temperatures above approximately 530 °C can be attributed to the change in the work function of SnO_2_. In such conditions, the work function of SnO_2_ becomes smaller than that of Ga_2_O_3_. This causes an outflow of the electric charge carriers from the SnO_2_ core to the Ga_2_O_3_ shell.

## 5. Conclusions

With the use of impedance spectroscopy, we investigated the impact of the Ga_2_O_3_ shell on the transfer of charge in the bulk and across the junction between SnO_2_/Ga_2_O_3_ core–shell nanobelts in ambient conditions. The bulk resistance of the SnO_2_/Ga_2_O_3_ core–shell nanobelts decreased due to the formation of a layer of accumulated electrons in the SnO_2_ core. At temperatures above approximately 530 °C, the thermal reduction of SnO_2_ and the associated decrease in its work function caused electrons to flow from the accumulation layer towards the Ga_2_O_3_ shell, which itself resulted in an increase in bulk resistance. The resistance of junctions in the as-prepared core–shell nanobelts was comparable to that in SnO_2_ nanobelts, as both structures are likely to be connected through existing SnO_2_/SnO_2_ homojunctions made of thin amorphous layers. In order to investigate the impact of the shell material on the modulation of the bulk and the junction resistance of core-shell structures, further systematical research incorporating reducing and neutral atmospheres is necessary. Expanding the range of the studied frequencies towards higher frequencies may help reduce the error in estimation of the bulk resistance.

## Data Availability

The original contributions presented in the study are included in the article/Supplementary Materials; further inquiries can be directed to the corresponding author.

## Supplementary Materials

The following supporting information can be downloaded at: https://www.mdpi.com/article/10.3390/s24196173/s1, Figure S1: Maps of the atomic composition of the cross-section of two SnO_2_/Ga_2_O_3_ core–shell fibrous structures with shells synthesized at 840 °C: (a) Ga; (b) Sn; (c) O; and (d) Pt.; Figure S2: Bode plots of the tested structures: (a) SnO_2_ nanobelts; (b) CS840; and (c) CS1000.

## References

[1] Ji H., Zeng W., Li Y. (2019). Gas sensing mechanisms of metal oxide semiconductors: A focus review. Nanoscale.

[2] Korotcenkov G. (2020). Current Trends in Nanomaterials for Metal Oxide-Based Conductometric Gas Sensors: Advantages and Limitations. Part 1: 1D and 2D Nanostructures. Nanomaterials.

[3] Comini E., Baratto C., Faglia G., Ferroni M., Vomiero A., Sberveglieri G. (2009). Quasi-one dimensional metal oxide semiconductors: Preparation, characterization and application as chemical sensors. Prog. Mater. Sci..

[4] Rashid T.-R., Phan D.-T., Chung G.-S. (2013). A flexible hydrogen sensor based on Pd nanoparticles decorated ZnO nanorods grown on polyimide tape. Sens. Actuators B Chem..

[5] Sysoev V.V., Schneider T., Goschnick J., Kiselev I., Habicht W., Hahn H., Strelcov E., Kolmakov A. (2009). Percolating SnO_2_ nanowire network as a stable gas sensor: Direct comparison of long-term performance versus SnO_2_ nanoparticle films. Sens. Actuators B Chem..

[6] Tonezzer M., Thi Thanh Le D., Van Duy L., Hoa N.D., Gasperi F., Van Duy N., Biasioli F. (2022). Electronic noses based on metal oxide nanowires: A review. Nanotechnol. Rev..

[7] Li T., Zeng W., Wang Z. (2015). Quasi-one-dimensional metal-oxide-based heterostructural gas-sensing materials: A review. Sens. Actuators B Chem..

[8] Long H., Li Y., Chai K., Zeng W. (2024). Metal oxide semiconductor-based core-shell nanostructures for chemiresistive gas sensing: A review. Sens. Actuators B Chem..

[9] Schipani F., Miller D.R., Ponce M.A., Aldao C.M., Akbar S.A., Morris P.A., Xu J.C. (2017). Conduction mechanisms in SnO_2_ single-nanowire gas sensors: An impedance spectroscopy study. Sens. Actuators B Chem..

[10] Park J.Y., Choi S.-W., Kim S.S. (2011). Junction-Tuned SnO_2_ Nanowires and Their Sensing Properties. J. Phys. Chem. C.

[11] Miao X.-Y., Zhu L.-Y., Wu X.-Y., Mao L.-W., Jin X.-H., Lu H.-L. (2023). Precise preparation of α-Fe_2_O_3_/SnO_2_ core-shell nanowires via atomic layer deposition for selective MEMS-based H_2_S gas sensor. Sens. Actuators B Chem..

[12] Kim J.-H., Kim S.S. (2015). Realization of ppb-Scale Toluene-Sensing Abilities with Pt-Functionalized SnO_2_–ZnO Core–Shell Nanowires. ACS Appl. Mater. Interfaces.

[13] Kim J.-H., Mirzaei A., Kim H.W., Kim S.S. (2018). Low power-consumption CO gas sensors based on Au-functionalized SnO_2_-ZnO core-shell nanowires. Sens. Actuators B Chem..

[14] Kim J.-H., Mirzaei A., Kim H.W., Kim S.S. (2020). Variation of shell thickness in ZnO-SnO_2_ core-shell nanowires for optimizing sensing behaviors to CO, C_6_H_6_, and C_7_H_8_ gases. Sens. Actuators B Chem..

[15] Park S., Ko H., Kim S., Lee C. (2014). Role of the Interfaces in Multiple Networked One-Dimensional Core–Shell Nanostructured Gas Sensors. ACS Appl. Mater. Interfaces.

[16] Singh N., Ponzoni A., Gupta R.K., Lee P.S., Comini E. (2011). Synthesis of In_2_O_3_–ZnO core–shell nanowires and their application in gas sensing. Sens. Actuators B Chem..

[17] Park S., Kim S., Sun G.-J., Lee C. (2015). Synthesis, structure and ethanol sensing properties of Ga_2_O_3_-core/WO_3_-shell nanostructures. Thin Solid Film..

[18] Raza M.H., Kaur N., Comini E., Pinna N. (2021). SnO_2_-SiO_2_ 1D Core-Shell Nanowires Heterostructures for Selective Hydrogen Sensing. Adv. Mater. Interfaces.

[19] Raza M.H., Kaur N., Comini E., Pinna N. (2020). Toward Optimized Radial Modulation of the Space-Charge Region in One-Dimensional SnO_2_–NiO Core–Shell Nanowires for Hydrogen Sensing. ACS Appl. Mater. Interfaces.

[20] Jang Y.-G., Kim W.-S., Kim D.-H., Hong S.-H. (2011). Fabrication of Ga_2_O_3_/SnO_2_ core–shell nanowires and their ethanol gas sensing properties. J. Mater. Res..

[21] Choi S.-W., Katoch A., Sun G.-J., Kim J.-H., Kim S.-H., Kim S.S. (2014). Dual Functional Sensing Mechanism in SnO_2_–ZnO Core–Shell Nanowires. ACS Appl. Mater. Interfaces.

[22] Alosfur F.K.M., Ridha N.J. (2021). Synthesis and characterization of ZnO/SnO_2_ nanorods core–shell arrays for high performance gas sensors. Appl. Phys. A.

[23] Choi S.-W., Katoch A., Kim J.-H., Kim S.S. (2015). Striking sensing improvement of n-type oxide nanowires by electronic sensitization based on work function difference. J. Mater. Chem. C.

[24] Hernández-Ramírez F., Tarancón A., Casals O., Arbiol J., Romano-Rodríguez A., Morante J. (2007). High response and stability in CO and humidity measures using a single SnO_2_ nanowire. Sens. Actuators B Chem..

[25] Huh J., Na J., Ha J.S., Kim S., Kim G.T. (2011). Asymmetric Contacts on a Single SnO_2_ Nanowire Device: An Investigation Using an Equivalent Circuit Model. ACS Appl. Mater. Interfaces.

[26] Keysight Technologies Impedance Measurement Handbook: A Guide to Measurement Technology and Techniques, 6th ed.; 2020; pp. 75–76. https://www.keysight.com/zz/en/assets/7018-06840/application-notes/5950-3000.pdf.

[27] Orazem M.E., Tribollet B. (2017). Electrochemical Impedance Spectroscopy.

[28] Costa I.M., de Araújo E.P., Arantes A.N., Zaghete M.A., Chiquito A.J. (2021). Unusual effects of nanowire-nanowire junctions on the persistent photoconductivity in SnO_2_ nanowire network devices. Nanotechnology.

[29] Li Q.H., Chen Y.J., Wan Q., Wang T.H. (2004). Thin film transistors fabricated by in situ growth of SnO_2_ nanobelts on Au/Pt electrodes. Appl. Phys. Lett..

[30] Malagù C., Carotta M.C., Fissan H., Guidi V., Kennedy M.K., Kruis F.E., Martinelli G., Maffeis T.G.G., Owen G.T., Wilks S.P. (2004). Surface state density decrease in nanostructured polycrystalline SnO_2_: Modelling and experimental evidence. Sens. Actuators B Chem..

[31] Prades J.D., Arbiol J., Cirera A., Morante J.R., Avella M., Zanotti L., Comini E., Faglia G., Sberveglieri G. (2007). Defect study of SnO_2_ nanostructures by cathodoluminescence analysis: Application to nanowires. Sens. Actuators B Chem..

[32] Kolmakov A., Zhang Y., Cheng G., Moskovits M. (2003). Detection of CO and O_2_ Using Tin Oxide Nanowire Sensors. Adv. Mater..

[33] King P.D.C., Lichti R.L., Celebi Y.G., Gil J.M., Vilão R.C., Alberto H.V., Piroto Duarte J., Payne D.J., Egdell R.G., McKenzie I. (2009). Shallow donor state of hydrogen in In_2_O_3_ and SnO_2_: Implications for conductivity in transparent conducting oxides. Phys. Rev. B.

[34] Mohamed M., Irmscher K., Janowitz C., Galazka Z., Manzke R., Fornari R. (2012). Schottky barrier height of Au on the transparent semiconducting oxide β-Ga_2_O_3_. Appl. Phys. Lett..

[35] Galazka Z. (2018). β-Ga_2_O_3_ for wide-bandgap electronics and optoelectronics. Semicond. Sci. Technol..

[36] Krawczyk M., Suchorska-Woźniak P., Szukiewicz R., Kuchowicz M., Korbutowicz R., Teterycz H. (2021). Morphology of Ga_2_O_3_ Nanowires and Their Sensitivity to Volatile Organic Compounds. Nanomaterials.

[37] Krawczyk M., Korbutowicz R., Szukiewicz R., Suchorska-Woźniak P., Kuchowicz M., Teterycz H. (2022). P-type Inversion at the Surface of β-Ga_2_O_3_ Epitaxial Layer Modified with Au Nanoparticles. Sensors.

[38] Varley J.B., Weber J.R., Janotti A., Van de Walle C.G. (2010). Oxygen vacancies and donor impurities in β-Ga_2_O_3_. Appl. Phys. Lett..

[39] Higashiwaki M., Sasaki K., Kamimura T., Hoi Wong M., Krishnamurthy D., Kuramata A., Masui T., Yamakoshi S. (2013). Depletion-mode Ga_2_O_3_ metal-oxide-semiconductor field-effect transistors on β-Ga_2_O_3_ (010) substrates and temperature dependence of their device characteristics. Appl. Phys. Lett..

[40] Rachut K., Körber C., Brötz J., Klein A. (2014). Growth and surface properties of epitaxial SnO_2_. Phys. Status Solidi.

