# Supplementary material for: Impedance Spectroscopy Study of Charge Transfer in the Bulk and Across the Interface in Networked SnO2/Ga2O3 Core–Shell Nanobelts in Ambient Air
Source: Sensors (Basel). 2024 Sep 24;24(19):6173. doi: 10.3390/s24196173 (PMC11478478; doi:10.3390/s24196173)
Supplement: Supplementary file 1 [file sensors-24-06173-s001.zip › sensors-3166470-supplementary.pdf]

Supplementary Materials

Impedance Spectroscopy Study of Charge Transfer in the Bulk and Across the Interface in Networked SnO_2/Ga_2O_3 Core–Shell Nanobelts in Ambient Air

Maciej Krawczyk *, Ryszard Korbutowicz and Patrycja Suchorska-Woźniak

Faculty of Electronics, Photonics and Microsystems, Wrocław University of Science and Technology, Wybrzeże Wyspiańskiego 27, 50-370 Wrocław, Poland; ryszard.korbutowicz@pwr.edu.pl (R.K.); patrycja.suchorska-wozniak@pwr.edu.pl (P.S.-W.)

* Correspondence: maciej.krawczyk@pwr.edu.pl

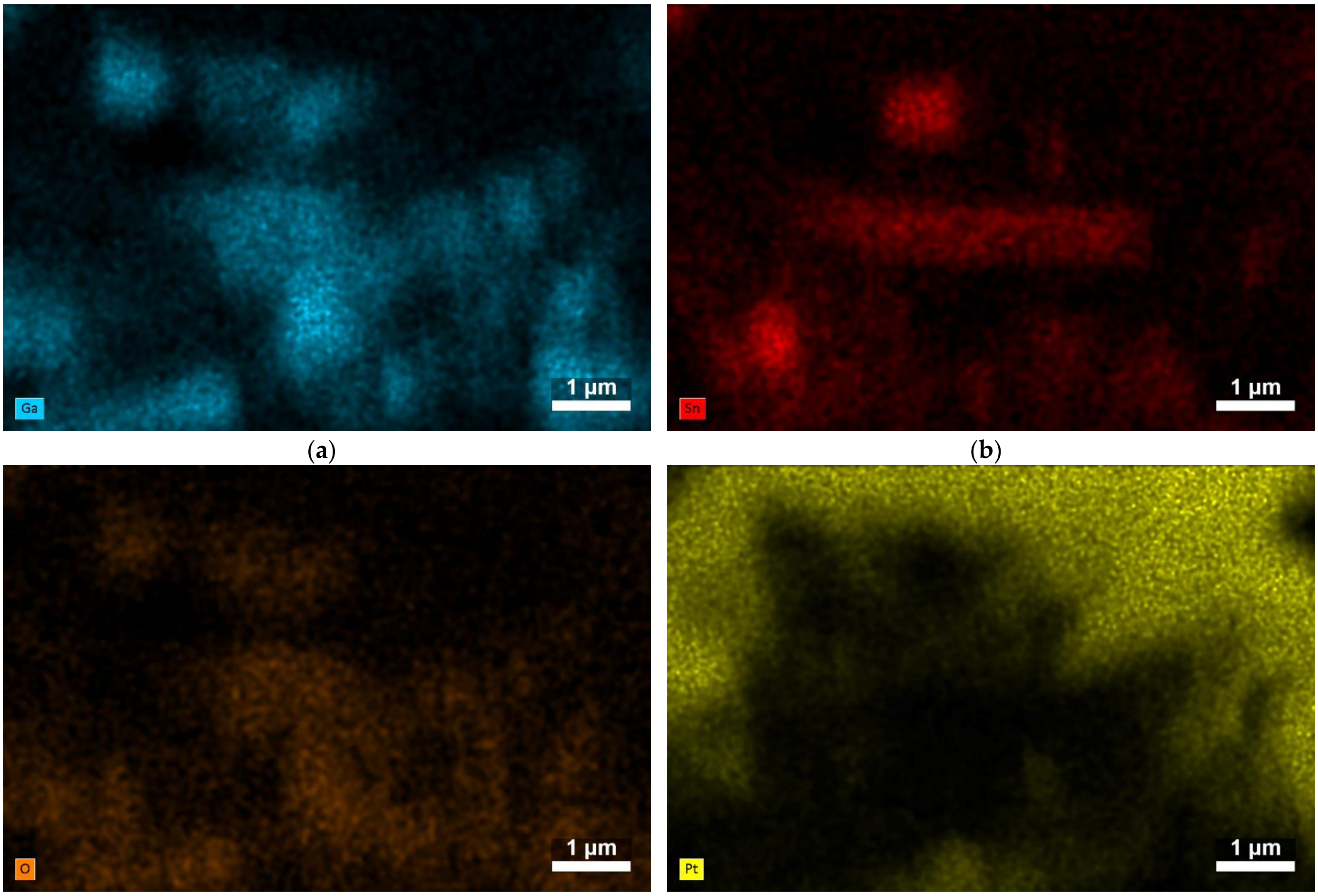

(**a**) (**b**) (**c**) (**d**)

Figure S1. Maps of the atomic composition of the cross-section of two SnO_2/Ga_2O_3 core–shell fibrous structures with shells synthesized at 840 °C: (**a**) Ga; (**b**) Sn; (**c**) O; (**d**) Pt.

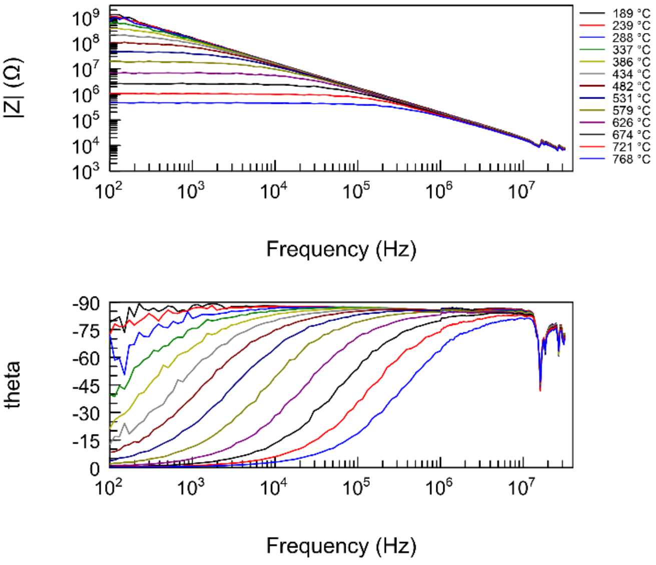

(**a**)

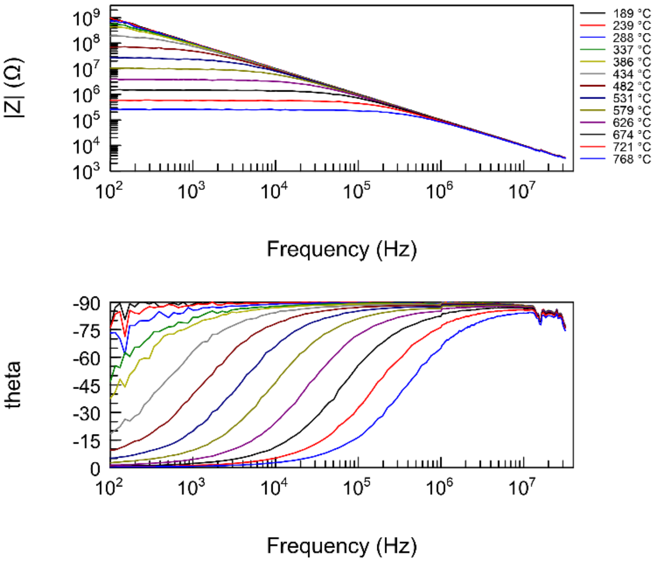

(**b**)

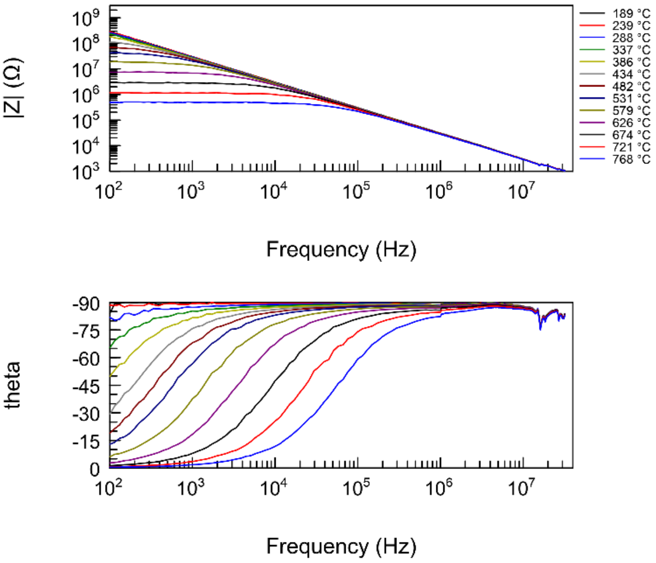

(**c**)

Figure S2. Bode plots of the tested structures: (**a**) SnO_2 nanobelts; (**b**) CS840; (**c**) CS1000.
